# The Role of Polycomb Group Protein BMI1 in DNA Repair and Genomic Stability

**DOI:** 10.3390/ijms22062976

**Published:** 2021-03-15

**Authors:** Amira Fitieh, Andrew J. Locke, Mobina Motamedi, Ismail Hassan Ismail

**Affiliations:** 1 Biophysics Department, Faculty of Science, Cairo University, Giza 12613, Egypt; fitieh@ualberta.ca; 2 Division of Experimental Oncology, Department of Oncology, Faculty of Medicine & Dentistry, University of Alberta, 11560 University Avenue, Edmonton, AB T6G 1Z2, Canada; alocke@ualberta.ca (A.J.L.); motamedi@ualberta.ca (M.M.)

**Keywords:** chromatin, genome instability, histone modification, transcription silencing, DNA repair, PcG, polycomb, PRC1, PRC2, BMI1, EZH2

## Abstract

The polycomb group (PcG) proteins are a class of transcriptional repressors that mediate gene silencing through histone post-translational modifications. They are involved in the maintenance of stem cell self-renewal and proliferation, processes that are often dysregulated in cancer. Apart from their canonical functions in epigenetic gene silencing, several studies have uncovered a function for PcG proteins in DNA damage signaling and repair. In particular, members of the poly-comb group complexes (PRC) 1 and 2 have been shown to recruit to sites of DNA damage and mediate DNA double-strand break repair. Here, we review current understanding of the PRCs and their roles in cancer development. We then focus on the PRC1 member BMI1, discussing the current state of knowledge of its role in DNA repair and genome integrity, and outline how it can be targeted pharmacologically.

## 1. Polycomb Group Proteins and PcG Protein Complexes

The Polycomb group (PcG) of proteins encode transcriptional repressors that play essential roles in maintaining stem cell pluripotency by repressing developmental genes [1,2,3]. These proteins were first discovered in the fruit fly *Drosophila melanogaster* as transcriptional regulators of key developmental genes known as homeobox (Hox) genes [4,5,6]. PcG proteins are highly conserved from *Drosophila* to human, and thus their function as transcriptional regulators of mammalian embryonic development and cell differentiation is well recognized [7,8], impacting the expression of genes important for cell fate decisions [9,10], embryogenesis [5,11,12,13], proliferation and stem cell self-renewal [14,15]. Of note, the balance between homeotic gene silencing and activation is maintained by another heterogeneous group of proteins called the trithorax group (TrxG) [16]. While PcG proteins mediate transcriptional repression, TrxG proteins counteract, activating expression of homeotic genes. Similar to PcG proteins, the TrxG proteins play vital roles in the epigenetic regulation of the cell cycle, senescence, DNA damage, and stem cell biology [17]. Together, the PcG and TrxG proteins control the cellular epigenetic memory system, which defines the set of modifications to a cell’s DNA that do not alter its sequence, and are inherited from the cell from which it descends [18].

**Figure 1:**
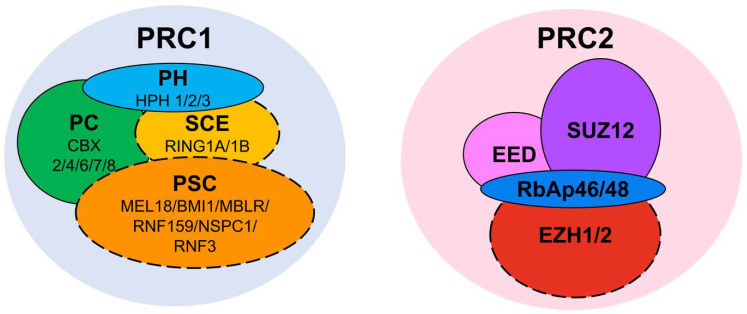
Major components of PRC1 and PRC2. Compositions of the two major types of the Polycomb repressive complexes. Mammalian PcG repressive complex 1 (PRC1) comprises interchangeable paralogs for the four main subunits: three of Polyhomeotic (PH), five of Polycomb (PC), two of Sex Comb Extra (SCE), and six of Posterior Sex Comb (PSC). Together, the SCE and PSC subunits contain the E3 ubiquitin ligase catalytic activity of PRC1 (dotted boundary). Human PcG repressive complex 2 (PRC2) consists of the core subunits EZH1/2, SUZ12, EED and RbAp46/48, with EZH1/2 (dotted boundary) possessing the methyltransferase catalytic activity of PRC2.

To regulate transcription, the PcG proteins form multimeric protein complexes called polycomb repressive complexes (PRCs). Two major PRCs have been characterized so far, PRC1 and PRC2 [19,20,21], and both alter chromatin to stably repress transcription at targeted genes [22,23] (Figure 1). Along with the maintenance of pluripotency, the PRCs are required for maintenance of stem cell proliferation; for instance, loss of PRC1 produces a severe defect in the proliferation of embryonic stem cells (ESCs) [24,25]. This control of cell proliferation is thought to occur through regulation of the *INK4b-ARF-INK4a* locus [26,27]. We will now turn our focus to the PRC complexes, describing PRC2 first and then shifting attention to PRC1.

## 2. PRC2

**Figure 2:**
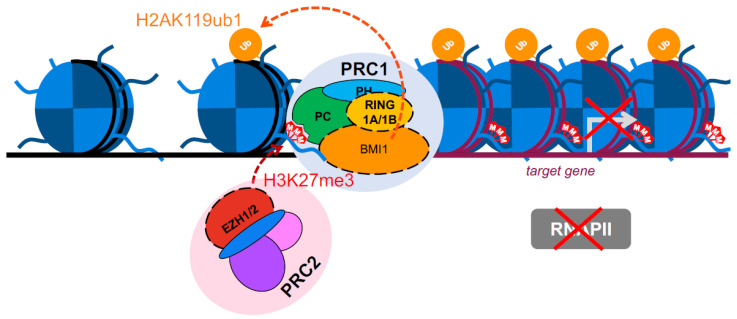
Traditional view of transcriptional repression mediated by PRC1 and PRC2. The enzymatic component of PRC2, EZH1/2, trimethylates histone H3 at lysine 27 (H3K27me3). PRC1 components recognize and bind to this modification and monoubiquitylate histone H2A at lysine 119 (H2AK119ub1). Ultimately, these modifications result in transcriptional repression of the target gene.

PRC2 consists of the core subunits Enhancer of Zeste Homolog 1 or 2 (EZH1/2), Suppressor of Zeste 12 protein homolog (SUZ12), Embryonic Ectoderm Development protein (EED), and Retinoblastoma protein Associated protein 46/48 (RbAp46/48) (in humans) (Figure 1). The enzymatic activity of PRC2 is to trimethylate histone H3 at lysine 27 (H3K27me3), generating a transcriptionally repressive epigenetic mark (Figure 2). This is carried out by the methyltransferase activities of the EZH1 or EZH2 subunits [21,28]. Although both proteins carry out the same enzymatic activity, they are thought to serve at specific times and contexts. EZH1 and EZH2 exhibit different expression patterns, with EZH1 being present in dividing and differentiated cells, and EZH2 only in actively dividing cells [29]. The EZH1-containing PRC2 complex also shows a lower H3K27 methyltransferase activity and distinct chromatin compacting properties relative to the EZH2-containing version [29]. It is important to note that the EZH2 subunit is inactive on its own and must be assembled with SUZ12 and EED to produce methyltransferase activity [23,30,31,32,33,34]. Beyond the core subunits, PRC2 can have alternate compositions, the core subunits interacting with a different array of proteins [35]. These proteins and their regulation by phosphorylation have been previously reviewed [36,37,38,39,40,41,42].

A genome-wide localization study showed that PRC2 binds to promoter regions, inducing methylation of H3K27 at the promoter chromatin of target genes including Hox genes and several other developmental regulators [43]. PRC2 may participate in a methylation cascade for gene repression; recently it was found that inhibition of protein arginine methyltransferase 5 (PRMT5), which catalyzes methylation of arginine residues on histones H3 and H4, prevents subsequent H3 methylation by PRC2, decreasing target gene expression [44]. Moreover, in AML (adult acute myeloid leukemia) cells, 50% of the genes downregulated by PRMT5 inhibition were partially rescued in expression upon inhibition of EZH2 activity, providing evidence of the critical role for PRC2-EZH2 in methylation and subsequent repression of these target genes [44]. Beyond methylation of H3K27, new reports are uncovering other non-canonical functions for the PRC2 components [45,46,47,48]. For example, EZH2 functions in prostate cancer cells in a manner independent of other PRC2 subunits, surprisingly acting as a transcriptional activator [49]. EZH2 was also shown to function in the constitutive activation of NF-κB target gene expression in ER-negative basal-like breast cancer cells, with this function independent of its histone methyltransferase activity [50]. Similarly, the EZH2 paralog EZH1 was associated with the transcriptionally active epigenetic mark H3K4me3, RNA polymerase II (RNAPII), and mRNA production [51]. These studies suggest PcG proteins may have additional roles different from their repressive functions in development.

PRC2 exists in two main forms, PRC2.1 and PRC2.2, and each has different subcomponents and function [52]. PRC2.1 contains Plant Homodomain Finger protein 1 (PHF1) and 19 (PHF19), Metal response element binding Transcription Factor 2 (MTF2), Elongin BC and PRC2 associated protein (EPOP), and PRC2 Associated LCOR Isoform 1/2 (PALI1/2). These components enable the complex to recognize unmethylated CpG islands in DNA [53,54]. In particular, knockout studies have shown that MTF2 mediates the *de novo* recruitment of PRC2.1 to unmethylated CpG islands of target genes [54]. The other form of PRC2, PRC2.2, contains AE Binding Protein 2 (AEBP2) and Jumonji and AT-Rich Interaction Domain containing 2 (JARID2) subunits [55]. While these subunits provide stability and conformation support to the entire PRC2 complex, AEBP2 and JARID2 also work in tandem to increase the catalytic activity of EZH2 [56,57,58,59]. Intriguingly, JARID2 has been noted for its affinity to monoubiquitylated lysine K119 on histone H2A (H2AK119ub1), recruiting PRC2.2 to the modification [60]. As discussed later, H2AK119ub1 is generated by the activity of PRC1, thereby suggesting an interplay between PRC1 activity and PRC2 recruitment (Figure 2).

## 3. PRC1

In fruit flies, PRC1 is typically composed of stoichiometrically equal amounts of Polycomb (PC), Posterior Sex Comb (PSC), Sex Comb Extra (SCE) and Polyhomeotic (PH). However, the mammalian PcG system is considerably more complicated, as each of the PRC1 components has multiple paralogs: five PC proteins (CBX2/4/6/7/8), six PSC (MEL18, BMI1, MBLR, RNF159, NSPC1 and RNF3), two SCE (RING1A and RING1B), and three PH (HPH1, HPH2 and HPH3) [61] (Figure 1). Notably, the PSC/SCE dimer component of PRC1 catalyzes the monoubiquitylation of histone H2A at lysine 119 (H2AK119ub1) [22,62], another epigenetic mark for transcriptional repression (Figure 2).

Recently, a comprehensive proteomic and genomic approach to characterize different mammalian PRC1 complexes was carried out [63]. By tandem affinity purification of PRC1 components, six distinct complexes were characterized (PRC1.1 - 1.6), each containing a different member of the PSC family. Furthermore, the RING1/YY1 (Yin and Yang 1)-binding protein (RYBP) was found to be part of PRC1 complexes; when this occurred in PRC1.2 and PRC1.4, the CBX proteins (chromobox proteins of the PC component), PHC (Polyhomeotic homolog) proteins, and the substoichiometric SCM (Sex Comb on Midleg) proteins, all canonical components, were excluded from the complex [63]. While it was originally thought that all PRC1 complexes are recruited to chromatin by the recognition of trimethylated histone H3 at lysine 27 (H3K27me3, generated by PRC2) via the chromo domain of the PC component (Figure 2), the observation that some PRC1 complexes can exist without PC proteins suggests other distinct recruitment mechanisms may exist (discussed below) [64,65,66,67]. Chromatin immunoprecipitation (ChIP)-sequencing analyses have also suggested the variant PRC1 complexes may have different chromatin targets and biological functions [63]. While the impact of different compositions in the activity of the PRC1 complexes largely remains to be determined, RYBP- and PC-containing versions of PRC1.4 can be reconstituted *in vitro*, and the RYBP-containing PRC1.4 has a distinct stimulatory effect on the enzymatic activity of RING1B-BMI1 [68]. Moreover, knockdown of RYBP results in the failure to ubiquitylate H2AK119, suggesting RYBP plays an important role in regulation of RING1B-BMI1 activity [63]. PC proteins have also been implicated in chromatin compaction [69]. In the absence of the PC component, however, RYBP-containing PRC1 complexes may be directing chromatin compaction via the affinity of RYBP for DNA [70], although the exact mechanism of this process is unknown.

The RING (really interesting new gene) finger protein genes were first identified in 1991 [71], and encode proteins that are the core components of the mammalian PRC1 system, RING1A (also known as RING1) and RING1B (also known as RING2) [72]. These two proteins, in complex with a PSC member (such as BMI1), form the E3 ubiquitin ligase that catalyzes the PcG-dependent ubiquitylation of histone H2A. RING1B has 336 amino acids and has 100% amino acid sequence conservation in human and mice. RING1A and RING1B share 49% amino acid sequence identity and share three conserved regions: the N-terminal RING finger involved in binding PSC proteins, and two regions at the C-terminus that form a globular domain involved in homodimerization [73]. Together, RING1A, RING1B, and BMI1 each contain a RING domain and embody the E3 ubiquitin ligase activity for PRC1. Of the three, RING1B is the major ubiquitin ligase for H2A, while BMI1 acts to stimulate the E3 activity of RING1B [22,74,75]. RING1A is a less efficient H2A ubiquitin ligase and thus is not the main H2A E3 ubiquitin ligase for PRC1 [76]. In agreement with this observation, mice lacking RING1A are viable but show skeletal deformities [77]. On the other hand, RING1B is essential during development, and mice lacking RING1B arrest in gastrulation and are embryonically lethal [78]. Inactivation of RING1B leads to a severe reduction in the global levels of H2A ubiquitylation [78]. However, H2A ubiquitylation on the inactive X chromosome is maintained in the absence of either RING1A or RING1B but not in cells lacking both, suggesting some overlap of function between RING1A and RING1B [74,79]. Recent studies show that inactivation of the catalytic activity of RING1B (Tamburri et al. (2020) using mutant RING1B at I53S [53] and Blackledge et al. (2019) at I53A/D56K) while retaining PRC1 assembly results in a complete loss of H2AK119 monoubiquitylation in vivo, subsequent displacement of PRC2 activity, loss of H3K27me3 deposition, loss of canonical PRC1 recruitment and loss of target gene repression in ESCs [80]. These findings indicate PRC1 catalytic activity is essential for Polycomb-mediated gene repression and chromatin domain interactions. Nevertheless, the relationship between H2AK119ub1 and transcriptional repression control is still heavily debated. Indeed, the combined action of PRC complexes could lead to transcriptional repression through ubiquitylation of histone H2A or by chromatin compaction independent of H2A ubiquitylation activity [2,81,82]. These processes can on their own or together lead to transcriptional repression by blocking the movement of RNAPII during elongation, recruiting complexes that repress transcription, or inhibiting the recruitment of complexes required for transcription [83,84,85,86,87]. More intriguingly, although histone H2A is the major substrate of the RING proteins, it is not the only target for ubiquitylation. The BMI1/RING1A duo act together to ubiquitylate DNA topoisomerase II⍺ for proteasomal degradation when cells are treated with the type II topoisomerase inhibitor etoposide [76], indicating RING1A could have hitherto undiscovered ubiquitylation targets.

Proteomic approaches have discovered various other complexes that contain some of the PRC1 complex proteins [88]. For instance, the E2F6.com-1 complex, which binds to and represses MYC and E2F6-responsive genes during the G0 stage of the cell cycle, contains several PcG proteins including RING1A, RING1B, MBLR (PCGF6), h-L3MBTL and YAF2 (YY1-Associated Factor 2) [89]. The BCoR repressor complex was also shown to contain the PRC1 proteins RING1A/B, RYBP and NSPC1, which localized to and repressed BCL6 gene targets [3]. These complexes raise the possibility that the RING1 proteins could have other functions not associated with PRC1.

## 4. Recruitment of PRCs to Target Loci

PcG proteins interact with chromatin as large protein-protein complexes, but the mechanism of their recruitment to chromatin remains enigmatic, since most PcG proteins do not bind DNA directly [90]. In *Drosophila*, *cis*-regulatory DNA elements, dubbed Polycomb Response Elements (PREs), are required for PcG recruitment. These PREs are thought to bridge PcG complexes to target genes by DNA-protein and protein-protein interactions [91]. In contrast to *Drosophila*, mammalian PcG complexes have a broad distribution across the target genes they repress, making it difficult to identify PREs and leaving the mechanism of PcG recruitment to chromatin unclear [92,93,94]. Association with specific DNA binding factors, binding to histone epigenetic marks, and interactions with non-coding RNAs are among several models suggested for the recruitment of PRC1 and PRC2 to their targets [21,95].

How might PRC2 be recruited to target loci? As discussed earlier, the MTF2 subunit of PRC2.1 mediates recruitment of the complex to unmethylated CpG islands [54]. On the other hand, PRC2 recruitment could also be mediated by Yin and Yang 1 (YY1), a ubiquitously expressed DNA binding zinc finger protein known to function as a sequence-specific transcriptional activator and repressor. Indeed, knockdown of YY1 in mouse myoblasts resulted in the dislodgement of PRC2 component EZH2 and the removal of H3K27me3 from target genes [96]. Alternatively, several reports implicate non-coding RNA (ncRNA) such as X-inactive specific transcript (Xist) RNA as a strong candidate for PRC2 recruitment [97,98]. In support of this, another study found that artificial tethering of long non-coding RNA (in this case, HOTAIR) to chromatin in the absence of PRC2 was sufficient to induce transcriptional repression [99]. This points to a possible mechanism in which rather than PRC2 recruitment being a mechanism to silence a target gene, PRC2 recruitment is a consequence of target gene silencing [99]. Finally, a recent study found that H2AK119ub1 may also mediate H3K27 methylation by regulating PRC2 recruitment [53], suggesting a dependence of PRC2 recruitment on PRC1 activity.

Turning to PRC1, in the canonical PRC1 complexes PRC1.2 and PRC1.4 (without RYBP), the chromodomain containing CBX proteins are known to play a role in recruitment of the PRC1 complex, as they recognize and bind H3K27me3 generated by PRC2, implying a dependence of canonical PRC1 on PRC2 activity [53,100]. Thus it was generally accepted that PRC1 is recruited to sites enriched with H3K27me3 by a PC protein binding the modification via its chromo domain [101]. However, the recent discovery of PRC1 complexes lacking the PC component [63], combined with the observation that PcG target genes are still ubiquitylated at H2AK119 in PRC2 null cells [65] suggests alternative mechanisms of recruitment exist. This is bolstered by additional evidence. The E3 ligase component of PRC1 in complex with RYBP can be detected at PcG target genes in PRC2 null cells [65], and chromatin binding of PRC1 is independent of the H3K27 methylation status of the targets [102]. As well, variant PRC1 complexes (PRC1.1, PRC1.3, PRC1.5, PRC1.6) that exclude the CBX protein component and contain RYBP or YAF2 do not recognize H3K27me3 and thus function independently of PRC2 [68,103,104]. Notably, a recent study demonstrated both RYBP and YAF2 bind H2AK119ub1 and subsequently recruit PRC1 to ubiquitylate H2A on neighbouring nucleosomes, indicating a positive feedback method of recruitment to target genes [105]. RYBP is also thought to recruit PRC1 to chromatin via binding to YY1 [106,107,108]. Still, genome-wide localization studies show binding of PRC1 at locations independent of YY1 (and PRC2) [109], and a recent study suggested the transcriptional repressor BCL6, in tandem with EZH2-dependent H3K27 trimethylation, could mediate the recruitment of non-canonical PRC1 complexes to their targets [110]. Clearly, the mechanisms of PRC1 and PRC2 recruitment to chromatin must involve myriad factors and interactions and will likely remain an active area of investigation in PRC biology.

## 5. PcG Proteins in Human Cancer

The identification of BMI1 as a proto-oncogene that cooperates with MYC to promote B and T-cell lymphomas [111,112] was the first evidence for the involvement of PcG genes in cancer development. BMI1 expression inhibits MYC-induced p19^ARF^-dependent apoptosis in part through suppressing the expression of the *INK4a/ARF* locus [113]. This locus encodes two tumour suppressors, p16^INK4a^ and p19^ARF^ in mice or p14^ARF^ in human, by differential splicing and the use of alternative reading frames [114]. p16^INK4a^ inhibits activation of the cyclin-dependent kinases (CDKs) CDK4/6 by preventing cyclin D from binding to CDK4/6. Inhibition of CDK4 and CDK6 activation allows the retinoblastoma protein pRb to repress E2F1-mediated transcription activity, resulting in G1 arrest [114]. While p19^ARF^/p14^ARF^ does not directly inhibit CDKs, it binds the p53 ubiquitin ligase MDM2, preventing MDM2 from inducing p53 proteasomal degradation [114,115]. Stabilized p53 subsequently inhibits cell cycle progression by inducing expression of the CDK inhibitor p21^Cip1^, and promotes apoptosis through a variety of mechanisms including the induction of BAX expression [116]. Overall, the effect of BMI1-dependent suppression of the *INK4a/ARF* locus results in the promotion of cell cycle progression and the inhibition of apoptosis.

Accumulating evidence demonstrates that BMI1 promotes tumorigenesis. For instance, in non-small cell lung cancer, BMI1 is required for K-Ras induced tumorigenesis in vivo and plays its tumorigenic role by repressing the *INK4b-ARF-INK4a* locus [117]. In addition, overexpression of BMI1 immortalized fibroblasts [113,118], and enhanced the expression of human telomerase reverse transcriptase (hTERT) in mammary epithelial cells, extending their replicative life span [119]. Other studies reveal BMI1 also adds a tumorigenic capacity in colon cancer [120], medulloblastoma [121], laryngeal cancer [122], breast cancer [123], prostate cancer [124], and pancreatic cancer [125]. Furthermore, BMI1’s oncogenic activity displays a relationship with PTEN (phosphatase and tensin homolog), a well-established tumour suppressor. It was reported that nuclear PTEN inhibits BMI1’s ability to suppress p16^INK4a^ and p19^ARF^/p14^ARF^, as well as its ability to upregulate hTERT in DU145 prostate cancer cells [126].

BMI1 is overexpressed in several cancers. Overexpression of BMI1 can cause neoplastic transformation and induce lymphomagenesis [111,127,128]. Elevation of BMI1 occurs in all primary myeloid leukemia [129] and mantle cell lymphomas [130]. Additionally, recent developments demonstrated the upregulation of BMI1 in human non-small cell lung cancer [131,132], breast cancer [123], colon cancer [123], human medulloblastomas [121], and prostate cancer [133,134,135]. Prostate cancer patients with the 11-gene signature of a stem cell-like expression profile, which contains BMI1, are more likely to have a short interval to disease recurrence, distant metastasis, and death after therapy [136]. As well, BMI1 protein was expressed at high levels in precancerous lesions of prostate cancer (prostatic intraepithelial neoplasias, PINs) and prostate carcinomas, and accompanied by reductions of p16^INK4a^ and p19^ARF^/p14^ARF^ [135]. Intriguingly, a study demonstrated that gene therapy targeting BMI1 can block chemoresistance in cancer cells, improving prognosis [137].

EZH2, a catalytic subunit of the PRC2 complex, is another PcG protein involved in cancer. EZH2 and BMI1 are highly expressed in glioblastoma [138,139,140,141]. The proliferation of glioblastoma cells could be blocked by downregulating EZH2 or BMI1 expression [138,141], further highlighting the role of both proteins in cancer development. EZH2 and BMI1 are similarly involved in lung tumour development, with EZH2 found highly expressed in squamous lung cell carcinoma together with BMI1 and the cellular proliferation marker Ki67 [142]. Several other tumours are characterized by a significant increase in both EZH2 and BMI1 expression, suggesting deregulation of PcG proteins is a common feature of transformed cells; such tumours include oral squamous cell carcinomas [143,144], hepatocellular carcinomas [145,146], gastrointestinal cancers [147,148,149], osteosarcomas [150], and bladder tumours [151,152,153,154].

Accumulating evidence indicates a strong connection between PcG proteins and prostate and breast cancers. EZH2 is involved in the progression of prostate cancer [155]. It has been identified as a direct downstream target of the pRB/E2F pathway and is one of the most frequently overexpressed genes in malignant prostate cancer [156]. Relatedly, EZH2 and BMI1 double-positive prostate carcinomas are associated with metastasis with an increasing likelihood of therapy failure and disease relapse [133]. The PcG proteins BMI1, RING1B, and CBX7, are also highly overexpressed in malignant prostate cancers [134,157]. This overexpression has been ascribed to deregulation of the pRB/E2F pathway, as well as specific amplification of BMI1 and EZH2 loci. Mechanistically, EZH2 overexpression promotes prostate cancer development by silencing ADRB2, a ß-adrenergic receptor; loss of ADRB2 expression induces cell invasion in benign prostate cells, whereas its constitutive expression counteracts the metastatic and proliferative effects induced by EZH2 overexpression. This regulatory pathway model is common to mammary epithelial cellular transformations [158]. Consistently, PcG proteins were also found highly overexpressed in breast cancer [156,159], and the high expression of EZH2 in preneoplastic mammary lesions suggests the deregulation of PRC2 activity is an early event in breast cancer development [160]. EZH2 was found essential for the proliferation of breast cancer type I susceptibility protein BRCA1^-/-^ cells [161], while EZH2 overexpression in breast cancer is associated with poor prognosis and correlates with metastatic sporadic and familial breast tumours [160,162]. BMI1 is also found overexpressed in breast cancers, and this correlates with MYC expression [163]. Additionally, BMI1 overexpression, in cooperation with H-Ras, caused the transformation of MCF10A breast epithelial cells [164,165].

The role of aberrant hypermethylation at genomic CpG islands has been extensively studied in various forms of cancers. Epigenetic profiling of human cancers has implicated the methylation of CpG islands in the transcriptional repression of tumour suppressor genes in cancers such as retinoblastoma, colorectal cancer, gastric carcinogenesis and renal carcinoma [166,167,168,169]. Although deregulated PRC2, via the overexpression or loss of function of EZH2, EED, or SUZ12, has been identified in numerous malignancies [170], the role of each form of PRC2 (PRC2.1 or PRC2.2) and the components they contain is not well characterized in these tumorigenic activities [170]. Moreover, concepts that require clarification include the ability of PRC2.1 to bind unmethylated CpG islands [171,172,173], the role of PRC2 in H3K27 methylation, the interplay between PRC1 and PRC2 activities, and the overall role of the PcG system in transcriptional repression. When solved, the answers to these gaps in research could have great potential in assisting the diagnosis and treatment of various cancers with hypermethylated CpG profiles.

## 6. BMI1 and the Enhancement of Double-Strand Break Repair

**Figure 3:**
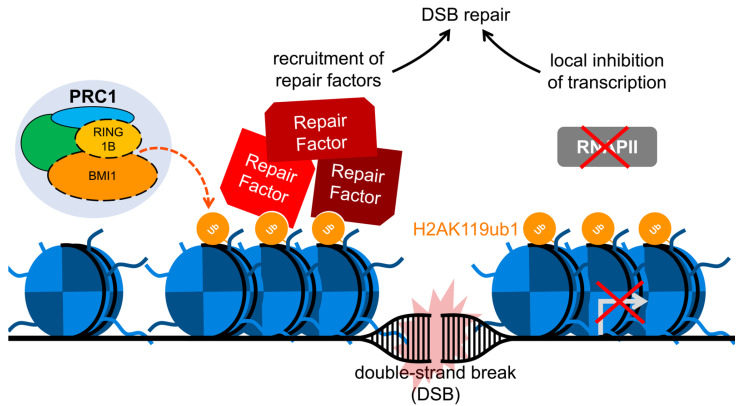
PRC1 in double-strand break repair. Double-strand break (DSB)-induced chromatin condensation upon ubiquitylation of histone H2A at K119 via the BMI1/RING1B E3 ubiquitin ligase component of PRC1. K119-monoubiquitylated H2A promotes the recruitment of repair factors as well as local transcriptional repression, both of which facilitate repair of the DSB.

In addition to its role in gene repression, histone H2A ubiquitylation is implicated as one important post-translational modification in the regulation of the DNA damage response (DDR). PRC1-dependent H2AK119 ubiquitylation is induced at sites of DNA damage and plays an essential role in recruiting repair factors during the DDR, possibly through chromatin remodeling to establish a barrier of silenced transcription at the sites of damage [174,175] (Figure 3). Accumulating evidence demonstrates critical functions of histone ubiquitylation in double-strand break (DSB) repair via the homologous recombination (HR) and non-homologous end joining (NHEJ) pathways. Indeed, the BMI1/RING1B E3 ubiquitin ligase was reported to contribute to DSB repair via histone H2A/H2AX ubiquitylation [176,177]. BMI1/RING1B also affects chromatin structure by generating H2AK119ub [22,74,75,178]. While H2AK119 ubiquitylation is a well demonstrated mechanism for suppressing gene transcription, further discussion will detail recent developments implicating the activity in facilitating DSB repair.

BMI1 was detected to rapidly recruit to DNA lesions caused by local micro-irradiation via ultraviolet laser, ionizing irradiation (IR), and the replication fork stalling agent hydroxyurea (HU) in a number of cell types, including U2OS osteosarcoma cells, mouse embryonic fibroblasts (MEFs), HeLa, and CD133^+^ glioblastoma multiforme (GBM) cells [176,179,180,181]. The recruitment of the BMI1/RING1B E3 ubiquitin ligase is required for the monoubiquitylation of γH2AX and H2A, likely at K119, at DSBs in U2OS cells and MEFs, as downregulation of BMI1 abolished the modification at those sites [176,177,182]. In support of H2A ubiquitylation being involved in DSB repair, BMI1 downregulation compromises the survival of U2OS, Hela, and GBM cells in response to IR treatment [176,177,180,181,183], in agreement with the notion that BMI1 facilitates DSB repair. Furthermore, BMI1-deficient MEFs display a two-fold reduction in the repair of DSBs induced by calicheamicin (CLM) at 5 h post-treatment, and the defects are rescued upon re-expression of BMI1 [176]. Similarly, knockdown of BMI1 reduced HR-mediated DSB repair [181] measured by an I-*Sce*l endonuclease-based in vivo HR assay in human embryonic kidney HEK293T cells. Moreover, BMI1 facilitated the recruitment of BRCA1 to laser stripes [176], and BRCA1 is essential for the commitment of cells to repair DSB using the HR pathway [184,185]. Intriguingly, there is also evidence to support a role of BMI1 in promoting NHEJ-mediated DSB repair, which requires the recruitment of 53BP1 (tumour protein p53 Binding Protein 1) to DSBs [184,186]. BMI1 enhances this recruitment and may physically interact with 53BP1 [138,176]. Direct evidence supporting a major role for BMI1/RING1B in NHEJ promotion was obtained in the context of dysfunctional telomere-initiated NHEJ, with depletion of either RING1B or BMI1 significantly reducing NHEJ-mediated telomere fusion [187]. However, RING1B deficiency in MEFs did not affect the repair of DSBs caused by gamma irradiation and only transiently decreased NHEJ-derived DSB repair at heterochromatin loci [187]. Nevertheless, in aggregate, the evidence supports the contributions of the BMI1-associated E3 ubiquitin ligase activity to DSB repair [176,188]. The involvement of the activity is supported by the requirement of BMI1’s RING finger domain in its recruitment to DSBs [176,181]. The RING finger domain mediates BMI1’s association with the catalytic subunit RING1B, and is therefore essential for BMI1-associated E3 ligase activity [106,189]. With this knowledge in mind, it will be worth determining whether re-expression of the RING finger-deleted BMI1 mutant is able to rescue the defects in repairing CLM-induced DSBs in BMI1^-/-^ MEFs. This observation will provide additional support that the observed rescue using wildtype BMI1 is attributable to its associated E3 ubiquitin ligase activity [176].

Structural analysis also supports the concept that the BMI1-associated ligase activity is important to facilitating DSB repair [190]. The DSB response protein RNF168 and BMI1/RING1B are both E3 ubiquitin ligases capable of conjugating ubiquitin to the nucleosome components H2A and H2AX. This activity is attributable to the positively charged residues R57 in RNF168 and K93 in RING1B, whereas the corresponding residue in another DSB response E3 ligase, RNF8, is a negatively charged residue D443, which inhibits this action [190]. Indeed, substituting R57 in RNF168 or K93 in RING1B with the negatively charged aspartate residue abolishes their abilities to ubiquitylate nucleosomal H2A/H2AX at K13/15 for RNF168 and K118/K119 for BMI1/RING1B [190]. On the substrate side, H2A/H2AX contains a nucleosome acidic patch (E61, D90, and E92) that is required for both E3 ubiquitin ligases to ubiquitylate their respective lysine residues [191,192]. In support of these observations, a recent crystal structure of BMI1/RING1B revealed that the structural interface of RING1B binding the H2A nucleosome acidic patch included residues R98, K93, and K97, with R98 being most critical [193]. RING1B R98 is positioned in the acidic pocket formed by E61, D90, and E92 of H2A, and contacts each of the acidic side chains [193]. In accordance with these observations, substituting RING1B R98 with an alanine residue led to a 50-fold decrease in nucleosomal ubiquitylation and a sharp reduction in RING1B’s affinity to nucleosomes [193]. In contrast to the dominant role of RING1B in PRC1’s association with the nucleosome, BMI1 does not significantly contribute to the nucleosome binding activity of PRC1 [193]. While it is likely that these structural mechanisms are also involved in BMI1/RING1B-associated ubiquitylation of H2A/H2AX at K118/K119 during the DDR, this has yet to be demonstrated experimentally.

Another question remains in what proportion of DSB repair is linked to BMI1-associated E3 ubiquitin ligase activity. It seems that a significant proportion occurs without a major contribution from BMI1; although in comparison to control cells, BMI1^-/-^ MEFs contain 2-fold more DSBs during a 5 h course of repair of CLM-induced DSBs, approximately 60% of DSBs in BMI1^-/-^ MEFs are repaired [176]. Will more DSBs be repaired in BMI1^-/-^ MEFs if sufficient time is given? This seems likely, as knockdown of BMI1 in U2OS and HeLa cells only modestly reduced cell survival in response to IR exposure [176,181]. In support of this, DSB repair in BMI1^-/-^ MEFs was only delayed compared to wildtype MEFs [187], and the same was reported in U2OS cells [194]. Alternatively, there might be factors waiting to be discovered that regulate BMI1-facilitated DSB repair. For example, the protein kinase AKT has been shown to phosphorylate BMI1, and this activity stimulates the BMI1-dependent accumulation of monoubiquitylated H2A at DSBs in MEFs treated with UV laser scissors, although not affecting BMI1 recruitment to the sites [195].

## 7. The Link Between DNA Damage and Transcriptional Repression

An emerging concept is the link between DNA damage and transcription. In support of a role for DSBs in chromatin relaxation and consequent transcriptional activation, several observations have emerged showing that stimuli-dependent, scheduled, or transient DNA damage across several physiological processes leads to transcriptional activation at specific promoters or enhancers. One of the first observations connecting DNA damage to transcriptional activation was DNA topoisomerase IIβ accumulation at the β-estradiol-sensitive pS2 promoter following hormone stimulation, along with proteins associated with the DDR such as PARP1, Ku, and DNAPK [196]. Here, the activity of topoisomerase IIβ induced transient DSBs that were necessary for transcriptional activation of the genes activated by the hormone. DNA damage-mediated transcription activation has also been reported in cultured primary neurons stimulated by various molecules that induce synapse-like signals, such as N-methyl-D-aspartate (NMDA), potassium chloride, and bicuculline [197]. Similarly in this case, transcription activation depended on transient DSBs induced by topoisomerase IIβ activity at gene promoters and enhancers, leading to RNAPII releasing from a paused state. Another report showed that after activation by dihydrotestosterone, DNA topoisomerase I-induced single stranded breaks (SSBs) at androgen-receptor binding sites activated enhancers and promoters in prostate cancer cells [198]. Here, the topoisomerase I-induced nicks were readily recognized and repaired by the DDR and DNA repair pathways, and knockout of the DDR or DNA repair pathways abolished androgen-induced transcriptional activation. Relatedly, in estrogen-sensitive breast cancer cells, hormone-induced gene activation is mediated by APOBEC3B activity. This enzyme is part of the AID (activation-induced cytidine deaminase)/APOBEC family and catalyzes the conversion of cytosine to uracil in DNA, a change recognized by the base excision repair (BER) pathway. Here, transient DSBs are generated to remove APOBEC3B-generated lesions and are required to recruit RNAPII to promoters [199]. Several models could explain the role of DNA damage-mediated transcriptional activation [200]. The simplest explanation is that DSBs are needed to release topological stress that accumulates in actively transcribed genes in order to promote RNA polymerase processivity; this is in agreement with the observations that topoisomerases are involved in most of the cases described here. Another hypothesis is that DSB repair is needed to induce chromatin reorganization and modifications that favour transcription, as was observed in the case of APOBEC3 [199].

In support of the DNA damage-dependent activation of transcription model, recent observations indicate noncoding RNAs are produced at the site of a DSB [201]. However, the converse idea, that DSBs have an overall inhibitory effect on pre-existing transcription, has also been known for a while [202,203,204]. This mechanism prevents aberrant transcripts from being produced at damaged DNA loci, which could alter cell homeostasis [205]. Recent reports have shown apparently contrasting data on the mechanism of DSB-dependent transcription inhibition. In particular, it was reported that generation of a DSB within a gene body resulted in transcription inhibition of the damaged gene in a DNAPK (DNA-dependent protein kinase)-dependent manner through the exclusion of RNAPII from the gene body and promoter [206]. However, this inhibition of transcription only affected the damaged locus and not the neighboring genes. This observation was recapitulated in vivo in mice through the genome-wide analysis of transcriptome alterations induced by the endonuclease I-*Ppo*I [204]. In contrast, a cluster of DSBs induced in an exogenous sequence repressed the activity of a distal promoter in *cis*, and in an ATM-dependent manner, through chromatin condensation, for several kilobases [202]. ATM was reported to promote H2A ubiquitylation, which in turn promoted PRC1 recruitment to DSBs. The contrasting views of these two reports might be explained by the peculiar features of the DNA loci where damage was induced; while Shanbhag and colleagues induced several breaks close to an exogenous sequence [202], Kim and coworkers generated single DSBs at endogenous loci mainly within ribosomal genes [204]. Interestingly, ATM-dependent transcription inhibition has also been reported for RNA polymerase I upon DNA damage induced in ribosomal DNA [207]. Although the effect of DNA damage on transcription inhibition is in apparent contrast with the observations of RNA-binding protein recruitment at DSBs along with de novo transcription stimulated by DDR signaling and DNA repair, it is possible that both phenomena occur [208,209,210,211]. It has been proposed that PARP (poly(ADP-ribose) polymerase)-dependent chromatin relaxation, required for the accessibility of DDR proteins at DSBs, might lead to the recruitment of RNAPII and transcription factors at sites of DNA damage [212]. This event may precede the ATM-dependent chromatin condensation required for further amplification of DDR signaling and transcription silencing.

Recent reports have focused on the potential transcriptional repression role of PRC1 at the DSB [202,213,214] (Figure 3). An initial report showed that ubiquitylated H2A is responsible for local chromatin condensation at the site of damage [202]. Later, BMI1 was revealed to have a major role in this process, as it interacts with transcriptional elongation complex ENL in the presence of DNA damage [214]. This interaction enables local transcriptional repression and, as proposed by Ui and colleagues, could promote accessibility of the damaged site to DNA repair factors [214]. However, it remains unclear how local transcriptional repression and formation of compact chromatin, normally promoted by PRC1, directly influences DNA repair. Most probably, other additional proteins interact with PRC1 components, facilitating the progression of DNA damage repair. Intriguingly, gene silencing at DSBs is a rapid and transient event, and can be promptly reversed upon completion of DSB repair [202]. This restoration of gene transcription at DNA lesions upon their repair also occurs in non-dividing cells in vivo, highlighting the wide physiological relevance of the phenomenon [204]. How transcription can be rapidly stopped and restarted in response to a DSB is still unclear. Whatever the mechanism driving the restoration of transcription, it now seems clear that a strict functional link with DNA repair must exist. In this regard, the deubiquitylating enzyme USP16 might be a noteworthy player, as it both terminates the ubiquitin signals downstream of RNF8/RNF168 during repair processes [215] and promotes transcription restoration at DSBs [202].

While DSBs can promote BMI1-dependent transcriptional silencing, there is evidence also for transcriptional silencing promoting DSB repair. A lack of gene silencing at DSBs in turn impedes DSB repair processes, as interfering with transcriptional repression at DSBs by preventing ATM-mediated phosphorylation of transcription elongation factor ENL or the chromatin remodeling complex PBAF results in increased sensitivity to ionizing radiation [194,214], decreased repair efficiency and an increased number of chromosome breaks [213,216]. It is worth noting that the role of PBAF in DNA repair activities becomes dispensable when ongoing transcription is inhibited prior to DNA damage, suggesting that PBAF involvement in DSB repair is related to its function in repressing transcription [213]. Similarly, delayed repair of DSBs is also an outcome of depleting PRC1 or PRC2 components, which have been recently identified as important factors in DNA repair in general [176,180,181,183,217,218]. Since these factors could participate in transcriptional repression at DSBs through chromatin compaction, it is conceivable that the condensed chromatin structure at DNA damage sites is no longer an obstacle to repair, but rather is actually required for proper DNA repair when the lesions occur next to or within a gene. It could be that the compacted chromatin state of the damaged locus may temporarily limit the mobility of DNA DSBs and prevent harmful translocations, suggested by the positive correlation between actively transcribed genomic regions and the propensity for translocations [219,220]. This reasoning is also supported by evidence that RNA templates may guide DNA synthesis during homology-directed DSB repair [221], suggesting that limiting the number of RNA transcripts from damaged DNA may be important to reduce undesired recombination events. Moreover, the resolution of DNA/RNA hybrid structures, which may potentially form as repair byproducts during DNA end resection when the DSB occurs within actively transcribed regions, has recently emerged as a key step for efficient DSB repair [222,223,224]. Altogether, these data demonstrate that transcriptional silencing of genes next to DSBs is an integral step of the DDR, contributing to efficient DNA repair when DSBs occur within actively transcribed regions.

## 8. A Role for PRC2 in Genomic Stability in Oocytes

Previously, elevated mRNA levels for PHF1, a core component of PRC2.1, were observed in human oocytes and in early pre-implantation embryos [225]. Relatedly, a recent study identified PHF1 as being required for the accurate alignment of chromosomes, oocyte ploidy, and regulating the asymmetric division of the mouse oocyte [225]. In a different series of experiments, EZH2 also played a role in chromosome alignment and euploidy in mouse oocytes [226]. They showed that EZH2 plays a role in regulating spindle assembly, extrusion of polar body I, and generally the meiotic maturation of mouse oocytes in coordination with BubR1 and p300/CBP Associated Factor (PCAF). Collectively, these findings place PcG proteins, and specifically PRC2, in a newly identified crucial role for oocyte meiosis. It is important to note that the findings were in the context of the mouse oocyte; a further examination of the role PcG proteins play in human oocyte development could be an avenue for future investigation.

## 9. Therapeutic Targeting of BMI1

BMI1 expression is frequently upregulated in numerous malignancies [137,227,228,229,230,231,232], and accumulating evidence indicate that BMI1 is involved in the maintenance of cancer stem-like cells (CSCs) [228,230]. Indeed, aberrant BMI1 expression is reported in many CSC contexts. BMI1 has been reported to be highly expressed in CD133^+^ murine liver CSCs and ovarian CSCs [231]. In addition, BMI1 was found to regulate the self-renewal of leukemic stem cells (LSCs) [129] and be involved in the regulation of CSCs from type-I neuroblastoma [233]. As well, in a mouse model of AML, BMI1-expressing LSCs were able to induce leukemia when transplanted into irradiated mice, whereas LSCs lacking BMI1 expression exhibited limited proliferative potential and were unable to induce leukemia, supporting the notion that BMI1 promoted the proliferation of LSCs [129]. The development of small molecule inhibitors against BMI1 will therefore pave the way for therapies that not only target highly proliferative cancer cells, but also the slowly dividing, therapy-evading cancer stem-like cells (CSCs). Additionally, targeting BMI1 within CSCs could potentially allow for modulation of two key tumour suppressor pathways driven by Rb and p53, as described earlier [234,235]. Moreover, a combinatorial approach utilizing BMI1 inhibitors with existing treatments might allow for the de-escalation of existing chemoradiotherapy protocols, minimizing associated toxicity and side effects.

Inhibition of BMI1 activity can be achieved through transcriptional, post-transcriptional, or post-translational means. For the route blocking transcription, broad spectrum histone deacetylase (HDAC) inhibitors have been shown to inhibit the expression of BMI1 and the activity of the PRC1 complex in breast cancer cells, as measured through a decrease of H1AK119ub1 [236,237]. However, due to their associated toxicity and side effects, HDAC inhibitor-mediated reduction of BMI1 expression is unlikely to be useful for cancer therapy. Another recent study isolated and identified a series of naturally occurring compounds targeting promoter activity at the BMI1 locus. The most active compound, wallichoside, decreased BMI1 protein levels in colon carcinoma cells and reduced the self-renewing capacity of human hepatocellular carcinoma cells [238]. Further in vivo studies in human-mouse xenograft models are warranted to provide a better understanding of the compound’s therapeutic value. In addition, the BMI1 RNA transcript can be targeted directly; molecules targeting the transcript have shown promising therapeutic potential in reducing the oncogenic potential of prostate CSCs [239] and hepatocellular carcinoma and liver CSCs [240]. Post-transcriptionally, BMI1 inhibition can be achieved by perturbing proteins that contribute to the normal regulation of BMI1. For instance, Polo-like kinase 1 (PLK1) is overexpressed in several cancer subtypes, correlates with poor patient outcomes, and plays an important role in driving tumour cell growth [241,242]. In breast cancer, small molecule inhibition of PLK1 resulted in the marked induction of cellular senescence [242,243]. Further experimentation revealed that downregulation of PLK1 activity caused upregulation of the microRNAs miR-200c and miR-141, which in turn post-transcriptionally inhibited expression of BMI1 [243].

Another way to modulate BMI1 function, localization and half-life is through post-translational modification [137]. Given the upregulation of BMI1 in CSCs and its essential role in promoting their proliferation, targeting BMI1’s post-translational modifications could present a more clinically relevant therapeutic modality with its potential to be selective for CSCs. The first suggestion of the imminent value of targeting BMI1 post-translationally came from a study where researchers observed fluctuating phosphorylation levels of BMI1 through cell cycle progression [244]. In G1/S phase, hypophosphorylated BMI1 was chromatin-bound, whereas the hyperphosphorylation of BMI1 in G2/M phase reduced its chromatin association. Later mechanistic insight into the kinase involved in BMI1 phosphorylation came from a yeast two-hybrid interaction assay, identifying the MAPK (mitogen-activated protein kinase)-activated protein kinase 3pK as a regulator of BMI1 chromatin association, among other PcG proteins [245]. In recent years, small molecule inhibitors developed by PTC Therapeutics (South Plainfield, NJ, US), have been designed to promote BMI1 hyperphosphorylation and hence its reduced chromatin association. These have been tested in colorectal carcinomas [246], lung adenocarcinomas [247], multiple myeloma [248], and prostate cancer [239]. In all cases, small molecule inhibition resulted in decreased BMI1 protein levels and reduced activity of the PRC1 complex. More importantly, diminished BMI1 levels reduced the self-renewal capacity of CSCs, which in turn correlated with lowered tumorigenic potential in vitro and in vivo [129,246]. As discussed previously, BMI1 represses the *INK4a/ARF* locus [113], thus loss of BMI1 should inhibit cell cycle progression and promote apoptosis. Aligning with these observations, cells treated with BMI1 inhibitors were more apoptotic [239,246,247,248] and underwent cell cycle arrest at G0 phase [246,247]. Collectively, these studies provide a strong rationale for including BMI1-targeted therapy in treatment strategies for patients with malignancies displaying elevated BMI1 expression. Another example of BMI1 post-translational modification is its βTrCP (beta-Transducin repeat Containing Protein)-mediated ubiquitylation and subsequent degradation [249]. Wildtype BMI1 is readily recognized and bound to by βTrCP, a subunit of the SCF (Skp1-Cullin F-box-containing) E3 ubiquitin ligase complex, and upon ubiquitylation, is destined for proteasome-mediated degradation [249]. Development of therapies increasing the extent of BMI1 ubiquitylation might present an avenue to continuously reduce BMI1 protein levels within the cell and thus ensure uninhibited transcription of the *INK4a/ARF* locus.

As shown, the targeting of BMI1 via several different mechanisms may provide therapeutic strategies for cancers that overexpress BMI1. Perhaps the emerging roles of PRC1 in DNA repair, beyond its established involvement in transcriptional repression, could broaden the uses of therapies targeting BMI1.
